# PAK6-mediated phosphorylation of PPP2R2C regulates LRRK2-PP2A complex formation

**DOI:** 10.3389/fnmol.2023.1269387

**Published:** 2023-12-18

**Authors:** Lucia Iannotta, Marco Emanuele, Giulia Favetta, Giulia Tombesi, Laurine Vandewynckel, Antonio Jesús Lara Ordóñez, Jean-Michel Saliou, Matthieu Drouyer, William Sibran, Laura Civiero, R. Jeremy Nichols, Panagiotis S. Athanasopoulos, Arjan Kortholt, Marie-Christine Chartier-Harlin, Elisa Greggio, Jean-Marc Taymans

**Affiliations:** ^1^Department of Biology, University of Padova, Padua, Italy; ^2^National Research Council, c/o Humanitas Research Hospital, Institute of Neuroscience, Rozzano, Italy; ^3^Univ. Lille, Inserm, CHU Lille, UMR-S 1172 - LilNCog - Lille Neuroscience & Cognition, Lille, France; ^4^Department of Pharmacology, Feinberg School of Medicine, Northwestern University, Chicago, IL, United States; ^5^University of Lille, CNRS, Inserm, CHU Lille, Institute Pasteur de Lille, US 41 – UAR 2014 – PLBS, Lille, France; ^6^IRCSS, San Camillo Hospital, Venice, Italy; ^7^Department of Pathology, Stanford University, Stanford, CA, United States; ^8^Department of Cell Biochemistry, University of Groningen, Groningen, Netherlands; ^9^YETEM-Innovative Technologies Application and Research Centre, Suleyman Demirel University West Campus, Isparta, Turkey; ^10^Centro Studi per la Neurodegenerazione (CESNE), University of Padova, Padua, Italy

**Keywords:** LRRK2, Parkinson’s disease, dephosphorylation, PP2A, PPP2R2C, Pak6, phosphorylation

## Abstract

Mutations in leucine-rich repeat kinase 2 (LRRK2) are a common cause of inherited and sporadic Parkinson’s disease (PD) and previous work suggests that dephosphorylation of LRRK2 at a cluster of heterologous phosphosites is associated to disease. We have previously reported subunits of the PP1 and PP2A classes of phosphatases as well as the PAK6 kinase as regulators of LRRK2 dephosphorylation. We therefore hypothesized that PAK6 may have a functional link with LRRK2’s phosphatases. To investigate this, we used PhosTag gel electrophoresis with purified proteins and found that PAK6 phosphorylates the PP2A regulatory subunit PPP2R2C at position S381. While S381 phosphorylation did not affect PP2A holoenzyme formation, a S381A phosphodead PPP2R2C showed impaired binding to LRRK2. Also, PAK6 kinase activity changed PPP2R2C subcellular localization in a S381 phosphorylation-dependent manner. Finally, PAK6-mediated dephosphorylation of LRRK2 was unaffected by phosphorylation of PPP2R2C at S381, suggesting that the previously reported mechanism whereby PAK6-mediated phosphorylation of 14-3-3 proteins promotes 14-3-3-LRRK2 complex dissociation and consequent exposure of LRRK2 phosphosites for dephosphorylation is dominant. Taken together, we conclude that PAK6-mediated phosphorylation of PPP2R2C influences the recruitment of PPP2R2C to the LRRK2 complex and PPP2R2C subcellular localization, pointing to an additional mechanism in the fine-tuning of LRRK2 phosphorylation.

## 1 Introduction

Parkinson’s disease (PD) is the second most common neurodegenerative disorder that affects 1–3% of the population, preferentially individuals over 65 years of age (Raza et al., 2019). Although several gene mutations can cause or predispose to PD, mutations in the gene encoding leucine-rich repeat kinase 2 (LRRK2) are among the most common. Indeed, over 40 missense mutations or risk factor variants in LRRK2 are linked to PD, accounting for ∼ 5% of all familial cases and 1% of all sporadic cases (Paisàn-Ruiz, 2009; Rocha et al., 2022). LRRK2-linked familial and sporadic PD share similar pathological manifestations suggesting that LRRK2 controls pathways crucial in both forms of the disease (Haugarvoll et al., 2008; Healy et al., 2008) as also reviewed in (Goveas et al., 2021). In addition, genome-wide association studies (GWAS) have identified genomic variants at the LRRK2 locus that confer risk for sporadic PD, suggesting that this gene is involved in the pathogenesis of a large portion of PD cases (Satake et al., 2009; Simón-Sánchez et al., 2009; Nalls et al., 2014, 2019). The LRRK2 gene encodes the LRRK2 protein, a large and complex serine/threonine kinase that, besides domains predicted to mediate protein-protein interactions, harbors a catalytic core constituted by the Ras-of-Complex (ROC) domain with a GTPase activity followed by the C-terminus of ROC (COR) domain and a kinase domain (Mata et al., 2006; Sejwal et al., 2017). The ROC-COR domain also mediates LRRK2 homodimerization that is crucial for LRRK2 to be active (Daniëls et al., 2011; Civiero et al., 2017; Watanabe et al., 2020; Myasnikov et al., 2021). The most frequent LRRK2 mutation, G2019S, sits in the kinase domain and results in increased kinase activity *in vitro* and in cells (Greggio, 2012). Other mutations located in the ROC-GTPase domain (R1441C/G/H and Y1699C), slow down GTP hydrolysis (Lewis et al., 2007; Daniëls et al., 2011; Taymans, 2012), which results in prolonged signaling and increased interaction of LRRK2 with its substrates (Steger et al., 2016). LRRK2 undergoes autophosphorylation at multiple sites *in vitro* and at S1292 in cells (Greggio et al., 2009; Sheng et al., 2012), and it is highly phosphorylated by other kinases particularly at a cluster of serines (S860, S910, S935, S955, S973, S976) located between the ANK and the LRR domains [reviewed in (Marchand et al., 2020)]. The phosphorylation level of these heterologous sites is crucial for the regulation of LRRK2 localization and function. Indeed, phosphorylated S910 and S935 are recognized by 14-3-3 proteins whose binding modulate LRRK2 subcellular localization and access to specific substrates (Dzamko et al., 2010; Nichols et al., 2010; Civiero et al., 2018; Iannotta et al., 2020). The balance between phosphorylated and non-phosphorylated forms of LRRK2 is tightly regulated by specific phosphatases. The phosphorylation levels of the phosphosites located in the ANK-LRR interdomain are regulated by protein phosphatases 1 and 2A (PP1 and PP2A) in multiple cell types. Indeed, an *in vitro* screen first identified PP1 as a LRRK2 phosphoregulator (Lobbestael et al., 2013) while an unbiased phosphatome-wide reverse genetics screen pointed to PP2A subunits as being involved in LRRK2 dephosphorylation (Drouyer et al., 2021). Previously, Athanasopoulos and colleagues identified PP2A as LRRK2 ROC domain interactor with the scaffolding subunit PPP2R1A and both PP2A catalytic subunits (Athanasopoulos et al., 2016). The dynamic binding of phosphatases to LRRK2 appears to be a key mechanism as association of specific PP1 and PP2A subunits to LRRK2 is indeed enhanced when LRRK2 is dephosphorylated at these heterologous phosphosites. This is the case after cellular or *in vivo* treatments with LRRK2 pharmacological inhibitors or for some pathogenic mutant forms of LRRK2 where steady state LRRK2 phosphorylation rates are reduced compared to wild type LRRK2 (Lobbestael et al., 2013; Marchand et al., 2020).

PP1 and PP2A denote dimeric and trimeric protein complexes, respectively, called holoenzymes that are responsible for about 90% of the phosphatase activity in eukaryotes. The active holoenzyme is constituted by a catalytic subunit (C) and a regulatory subunit (B) (and a scaffolding subunit (A) in the case of PP2A) that is responsible for the binding with the specific substrate. Mammals display three different PP1-catalytic subunits (PPP1CA, PPP1CB, and PPP1CC) and at least 150 regulatory subunits which result in at least 450 possible heterodimeric holoenzyme combinations (Bollen et al., 2010). On the other hand, there are more than 100 heterotrimeric holoenzyme compositions of PP2A, which include a catalytic subunit (either PPP2CA or PPP2CB), a regulatory subunit and a scaffolding module (Wlodarchak and Xing, 2016). The key to understanding the specificity of LRRK2 dephosphorylation by PP1 or PP2A therefore lies in the identification of the regulatory subunits involved. While the precise PP1 regulatory subunit remains elusive, our recent study identified the PP2A complexes PPP2CA:PPP2R2A/B/C/D as the phosphatase holoenzymes active on LRRK2 ANK-LRR interdomain phosphosites (Drouyer et al., 2021). Intriguingly, PP2A-mediated LRRK2 dephosphorylation is the signal that leads to LRRK2 ubiquitination and subsequent degradation (Drouyer et al., 2021).

A few years ago, through another unbiased screen, we identified the p21-activated kinase 6 (PAK6) as a *bona fide* LRRK2 interactor (Beilina et al., 2014; Civiero et al., 2015). PAK6 is a member of the PAK family, which has been implicated in different cellular mechanisms including cytoskeleton remodeling, cell motility, modulation of gene expression as well as inflammatory responses or apoptosis (Civiero and Greggio, 2018). In mammals, PAK family consists of six serine/threonine kinases subdivided in two groups: Group I (PAK 1-2-3) and Group II (PAK 4-5-6). PAKs were initially described as effectors of small GTPases of Rho family, namely, Cdc42 and Rac1, but more recently a broad range of new interactors has been discovered. In group II PAKs the binding with small GTPases at the Cdc42/Rac1 binding domain (CRIB) is crucial for the re-localization of the kinase within specific subcellular compartment (Baskaran et al., 2012). The CRIB domain also mediates the binding between PAK6 and the ROC-GTPase domain of LRRK2, allowing the two proteins to cooperate in controlling neurite complexity in mammalian brain (Civiero et al., 2015). Notably, a constitutively active form of PAK6 was found to rescue the G2019S LRRK2-associated neurite shortening phenotype in BAC mice overexpressing murine Lrrk2-G2019S, via phosphorylation of 14-3-3γ (Civiero et al., 2017). Of note, PAK6-mediated phosphorylation of 14-3-3γ results in loss of affinity between LRRK2 and 14-3-3γ with consequent 14-3-3 release and LRRK2 dephosphorylation at S935 (Civiero et al., 2017). Together these findings suggest a possible relationship between PAK6, 14-3-3 binding and phosphatases in regulating LRRK2 phosphorylation levels.

Here, we hypothesized that PAK6 may functionally interact with and phosphorylate LRRK2 phosphatases and thereby influence their ability to bind and dephosphorylate LRRK2. To this test this hypothesis, we focused on those phosphatase subunits that we have previously found to be involved in LRRK2 dephosphorylation (Drouyer et al., 2021).

## 2 Materials and methods

### 2.1 Plasmids

Eukaryotic expression constructs of GFP-LRRK2 WT and 2xMyc-PAK6 WT were obtained from Addgene (Cambridge, MA, USA). The pLV-CSJ-3xFlag-LRRK2 plasmids were available in the laboratory (Drouyer et al., 2021). PAK6 mutant variants S531N and K436M were generated using the Quick-Change II site-directed mutagenesis kit (Stratagene) as previously described (Civiero et al., 2015). Eukaryotic expression constructs for 3xFlag-tagged or GFP-tagged phosphatases, including PPP1CA, PPP2CA, PPP2CB, PPP2R2A, PPP2R2B and PPP2R2C, wild type and mutants forms, were generated via the cloning services of e-Zyvec (Lille, France).

### 2.2 Cell culture and transfection

HEK293T cells were cultured in Dulbecco’s modified Eagle’s medium supplemented with 10% fetal bovine serum, 100 U/ml penicillin, 100 μg/ml streptomycin and 2.5% HEPES. Cells were plated in 12-well plates or 10-cm of 15-cm culture dishes and transfected at 80% of confluence with plasmid DNA using polyethylenimine (Polysciences) according to the manufacturer’s recommendations.

### 2.3 Protein purification and *in vitro* phosphorylation

Flag-tagged proteins were purified as described previously (Civiero et al., 2012; Drouyer et al., 2021). In brief, HEK-293T cells were cultured in 15-cm dishes and transfected at 70–80% confluence with Flag-tagged PAK6 or Flag tagged phosphatases subunits. Lysates were collected 48 h after transfection in 500 μl of lysis buffer (20 mM Tris-HCl pH 7.5, 150 mM NaCl, 1 mM EDTA, 1% Triton, 10% glycerol) containing protease and phosphatase inhibitor cocktail (Thermo Fisher Scientific)], incubated for 30 min at 4°C on a rotary wheel and clarified by centrifugation at 14,000 g, 10 min at 4°C. The supernatants were then incubated for 2 h with constant rocking at 4^°^C with anti-Flag-M2-agarose beads which were then washed four times in washing buffer (25 mM Tris-HCl pH 7.5, 400 mM NaCl, 1% Triton) and two times in phosphorylation buffer (25 mM Tris pH 7.5, 10 mM MgCl_2_, 2 mM DTT, 0.02% Triton, 5 mM b-glycerophosphate) supplemented with protease inhibitor cocktail (Roche). Proteins were eluted in phosphorylation buffer containing 1 mM DTT and 100 μg/ml of 3xFlag peptide, for 15 min at 4°C with constant rocking.

For *in vitro* phosphorylation, purified LRRK2 and purified phosphatases eluted in the *in vitro* phosphorylation buffer, were mixed and incubated for 60 min at 30°C with 100 μM ATP. Samples were then submitted to PhosTag analysis or mass spectrometry-based phosphosite mapping as described below.

### 2.4 Co-Immunoprecipitation and western blotting

After transfection with plasmid DNA, cells were further cultured for 48h at 37°C, 5% CO_2_. The cells were then rinsed in 1X PBS and harvested in 1 mL of buffer A (20 mM Tris-HCl pH 7.4, 150 mM NaCl, 10 mM MgCl_2_, 0.1% Triton, 10% Glycerol) containing Proteases/phosphatase inhibitors (PhosSTOP Sigma-Aldrich; complete ™ Protease Inhibitor Cocktail Roche) or RIPA buffer (20 mM Tris–HCl pH 7.5, 150 mM NaCl, 1mM EDTA) containing 1% protease inhibitor cocktail (Sigma-Aldrich) and phosphatase inhibitors (2.5 mM sodium pyrophosphate, 1mM β-glycerophosphate, and 1 mM sodium orthovanadate). Lysates were incubated for 30 min at 4°C then centrifuged for 30 min at 15,000 *g* and the supernatant recovered.

For co-immunoprecipitations, beads were washed twice with the lysis buffer A and 950 μL of lysate (50 μL are put aside to test input levels) was incubated end-over-end with GFP-TrapA beads (ChromoTek) or anti-Flag M2 agarose beads for 2 h or overnight at 4°C. Immune complexes were incubated at 95°C for 10 min in LDS sample buffer and then loaded on gels. Protein content of cell lysates was determined using the bicinchoninic acid (BCA) protein determination assay (Pierce Biotechnology) or the Bradford method (Thermo Scientific) with bovine serum albumin (BSA) as the standard. A total of 30 μg cell lysates were resolved by electrophoresis on NuPAGE 3–8% Tris-Acetate gradient gels, 4–12% Bis-Tris gradient gels, 4–12% Tris-Glycine gradient gels or 12.5% SDS gels (LifeTechnologies) or ExpressPlus PAGE precast gels 4–20% (GeneScript). Separated proteins were transferred to PVDF (Bio-Rad) or nitrocellulose (Amersham) membranes, and non-specific binding sites were blocked for 1h in Tris-buffered saline containing 0.1% Tween-20 (TBS-T) and 5% non-fat milk or 5% BSA. Membranes were then incubated overnight at 4°C with the appropriate antibodies: rabbit anti-β-tubulin (ab6046; Abcam, 1:30,000), mouse anti-Flag-HRP (A8592; Sigma-Aldrich, 1:5000 or 1:50000), rabbit anti-LRRK2 (MJFF2 c41-2; Abcam, 1:300), rabbit anti-pS935 LRRK2 (ab133450; Abcam, 1:300), rabbit anti-pS1292 LRRK2 (Abcam, ab203181, 1:1000), rabbit anti-GFP (Invitrogen, A11122, 1:1000), mouse anti-myc tag (Millipore, 05-724, 1:2000), mouse anti-Flag M2 (Sigma Aldrich, F1804, 1:500), rabbit anti-pT73 RAB10 (Abcam, ab230261, 1:1000), mouse anti-RAB10 (ThermoScientific, MA515670, 1:1000), Rabbit anti-GAPDH (Sigma-Aldrich, G9545, 1:5000) and mouse anti-c-myc-Peroxidase (11814150001; Roche; 1:2000). Blots were rinsed three times with TBS-T and incubated for 1h at RT with the appropriate HorseRadish-Peroxidase (HRP)-conjugated secondary antibodies (Invitrogen). The bands were visualized using Immobilon^®^ Forte Western HRP Substrate (Millipore) or LI-COR dual probes and the VWR^®^ Imager Chemi Premium. Images were acquired and densitometric analysis were performed using Aida analyzer v1.0 (Raytest), image analyzer ImageQuant 600 (GE Healthcare Bio-Sciences) and ImageJ.

### 2.5 PhosTag assay

Samples were mixed with 4X Laemmli’s SDS-PAGE sample buffer and heated at 95°C for 5 min. Samples were separated on to a PhosTag gel (SuperSep PhosTag TM 50 uM, 15% and 17 well, Wako, Osaka, Japan) with WIDE-VIEW Pre-stained Protein Size Marker (Osaka, Japan) with WIDE-VIEWTM 50ted at 953 × 10 min in the transfer buffer containing 10 mM EDTA followed by one time 10 min wash in the transfer buffer without EDTA. The gels were transferred to nitrocellulose membrane at 5 V overnight with ice-cold transfer buffer at 4°C or at 50 V for 1 h 45 with transfer buffer at room temperature (RT). Membranes were revealed in the same way as normal western blot membranes.

### 2.6 SDS-PAGE, Coomassie staining and mass spectrometry-based phosphosite mapping for purified proteins

Purified phosphatases were resolved by electrophoresis on NuPAGE 4–12% Tris-Glycine gradient gels (LifeTechnologies). Gels were submitted to Coomassie staining according to the manufacturer’s protocol (PageBlue protein staining solution, Thermo Fisher).

Spots were excised from stained gels and tryptic digestion was performed as previously described (Reyniers et al., 2014). An Ultimate 3000 RSLC nano System (Thermo Fisher Scientific) was used for the separation of protein digests. Peptides were automatically fractionated onto a commercial C18 reverse phase column (75 μm × 150 mm, 2-μm particle, PepMap100 RSLC column, Thermo Fisher Scientific) at 35°C. Trapping was performed during 4 min at 5 μL/min, with solvent A (98% H2O, 2% acetonitrile-ACN and 0.1% formic acid-FA). Elution was performed using two solvents, A (0.1% FA in water) and B (0.1% FA in ACN) at a flow rate of 300 nL/min. Gradient separation was 2 min from 2 to 5% B, 12 min from 5 to 25% B, 2 min from 25 to 80% B, 3 min 80% B. The column was equilibrated with 2% buffer B prior to the next sample analysis. The eluted peptides from the C18 column were analysed by a Q-Exactive device (Thermo Fisher Scientific). The electrospray voltage was 1.9 kV, and the capillary temperature was 275°C. Full MS scans were acquired in the Orbitrap mass analyzer over m/z 300–1,200 range with a resolution of 35,000 (m/z 200). The target value was 5.00E + 05 and the maximum allowed ion accumulation times were 250 ms. Three most intense peaks with charge state between 2 and 4 were fragmented in the HCD collision cell with normalized collision energy of 35%, and tandem mass spectra were acquired in the Orbitrap mass analyser with a resolution of 17,500 at m/z 200. The target value was 5.00E + 0.4 and the maximum allowed ion accumulation times were 150 ms. Dynamic exclusion was set to 7 s.

Raw data collected during nano-LC-MS/MS analyses were processed and converted into *. mgf peak list format with Proteome Discoverer 1.4 (Thermo Fisher Scientific). MS/MS data were interpreted using search engine Mascot (version 2.4.0, Matrix Science, London, UK) Searches were performed with a tolerance on mass measurement of 0.2 Da for the precursor and 0.2 Da for the ion fragment, against two composite target decoy databases built with Homo Sapiens Swissprot databases (TaxID = 9606, April 10 2017, 20,173 entries) fused with the sequences of recombinant protein PPP2R2C, trypsin and a list of classical contaminants (118 entries). Cysteine carbamidomethylation, methionine oxidation, protein N-terminal acetylation, cysteine propionamidation, serine, threonine, arginine, tyrosine and lysine phosphorylation were searched as variable modifications. Up to four trypsin missed cleavage was allowed.

### 2.7 Immunocytochemistry

HEK293T cells were transfected and fixed after 24h using 4% paraformaldehyde (PFA)/PBS for 20 min at RT. Then, cells were permeabilized in 0.1% Triton X-100/1X PBS for 20 min at RT and blocked with 5% v/v FBS in 1X PBS for 60 min at RT. Primary antibodies incubation was performed using mouse anti-FlagM2 (Cat #F1804; Sigma-Aldrich, 1:400) and rabbit anti-PAK6 (Cat #ab154752; Abcam, Cambridge, UK, 1:200). Secondary antibodies anti-mouse Alexa Fluor 488 (A11029, Invitrogen) and anti-rabbit Alexa Flour 568 (A11036, Invitrogen) fluorophores were diluted 1:200 in 5% v/v FBS in 1X PBS and incubated for 1h at RT. Nuclei were counterstained with Hoechst 1:10,000 and mounted on a glass microscope slide (ThermoFisher) using Mowiol.

### 2.8 Proximity ligation assay

Proximity ligation assays (PLA) were performed on HEK293T cells transfected with GFP-LRRK2 WT, 3xFlag-PPP2R2C WT or S381A, 3xFlag-PPP2CA in presence or absence of 2xMyc-PAK6 S531N following the manufactured instructions. Briefly, after fixing for 20 min at RT with 4% PFA and permeabilizing with 0.1% Triton X-100/1X PBS for 20 min at RT, sample were blocked for 1h at RT with blocking solution (5% FBS in 1X PBS). Rabbit LRRK2 MJFF2 (c41-2) (Abcam, Cambridge, UK, Cat #ab133474, 1:300) and mouse Flag M2 (Sigma-Aldrich, Cat #F1804, 1:300) were diluted in blocking solution and incubated overnight in humidity chamber at 4°C. The day after, samples were incubated with PLA probes (Cat # DUO92002 and DUO92004, Sigma-Aldrich) diluted in blocking buffer for 1h at 37°C and washed two times for 5 min with 1X Buffer A. The pre-diluted Ligation-Ligase solution (Cat# DUO92007, Sigma-Aldrich) (diluted 1:40 in blocking buffer) was added to samples and then incubated for 30 min at 37°C. After washing two times for 2 min with 1X Buffer A, the incubation with the pre-mixed Amplification-Polymerase solution was carried out for 100 min at 37°C. Finally, cells were washed two times with 1X wash Buffer B for 10 min each, one time with 0.01X Wash Buffer B for 1 min, incubated with Hoechst 33258, pentahydrate (Cat #H3569, Invitrogen) for 5 min at RT and then mounted with Mowiol for the subsequent imaging analysis. Images were acquired at the confocal microscopy Zeiss LSM700, with a 63X oil-immersion objective and analyzed with ImageJ software.

### 2.9 Statistical analysis

Statistical analysis has been performed using GraphPad Prism 9.5. Quantitative data are expressed as mean ± SEM (standard error of the mean) and represent at least three independent sets of experiments. Significance of differences between two groups was assessed by Student t-test or one sample t test and by one-way ANOVA or two-way ANOVA followed by Tukey’s *post-hoc* test when more than two groups were compared.

## 3 Results

### 3.1 PAK6 phosphorylates the PP2A regulatory subunit PPP2R2C

**FIGURE 1:**
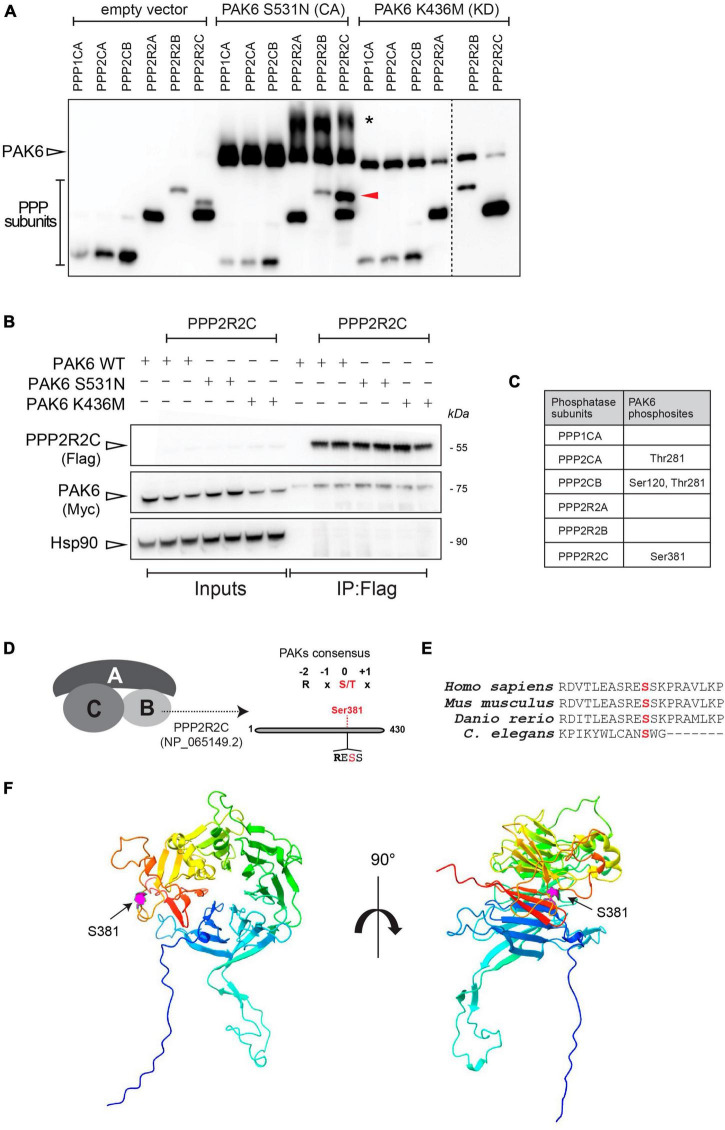
PAK6 phosphorylates PPP2R2C, PPP2CA and PPP2CB *in vitro*. **(A)** PhosTag blot of purified phosphatases subunits were incubated without PAK6 or with PAK6 constitutively active form S531N or kinase dead K436M. An additional phosphorylation band appears for PPP2R2C incubated with PAK6 S531N but not with the kinase dead PAK6 K436M. **(B)** Co-immunoprecipitation to test binding of PAK6 with PPP2R2C was performed as described in materials and methods, using 3xFlag-PAK6 as bait. Subsequent western blot analysis of the captured complexes confirms binding of PAK6 to PPP2R2C. **(C)** Summary table of results from phosphosite enrichment and MS analysis of *in vitro* phosphorylation reactions of different phosphatase subunits with PAK6 showing that S381 on PPP2R2C is a PAK6 phosphosite. **(D)** Schematic of PAK6 phosphorylation on PPP2R2C. **(E)** Multiple alignment of PPP2R2C (isoform a) in vertebrate mode organisms and in *C. elegans*. **(F)** PPP2R2C 3D reconstruction. The AlphaFold structure of PPP2R2C (reference AF-Q9Y2T4-F1) is depicted with rainbow coloring (blue corresponding to the N-terminus and red to the C-terminus), with magenta highlighting of the Ser381 residue that is phosphorylated by PAK6. The left panel shows the 7-bladed WD40 propeller structure in a frontal view and the right panel is the same model rotated 90° around the y-axis.

Since PAK6 kinase activity induces dephosphorylation of LRRK2 at Ser935 (Civiero et al., 2017), we set out to test whether this is due to a direct phosphorylation of LRRK2’s phosphatases by PAK6, in addition to a mechanism involving PAK6 phosphorylation of 14-3-3s (Civiero et al., 2017). To this aim, we performed a kinase assay using recombinant 3xFlag-PAK6 S531N (constitutively active) and K436M (kinase dead) and different LRRK2’s phosphatases subunits as substrates, namely, 3xFlag-PPP1CA (PP1 catalytic subunit), 3xFlag-PPP2CA and PPP2CB (PP2A catalytic subunits), 3xFlag-PPP2R2A, PPP2R2B and PPP2R2C (PP2A regulatory subunits) (Supplementary Figures 1, 2). To evaluate whether PAK6 can phosphorylate any of these subunits, we employed PhosTag gels, which allow to separate pools of non-phosphorylated versus phosphorylated proteins based on their different migration properties upon PhosTag binding. We found that PAK6 S531N can efficiently phosphorylate the PP2A regulatory subunit PPP2R2C in vitro, as shown by the presence of an upper band of PPP2R2C when incubated with PAK6 S531N but not with kinase dead PAK6 (Figure 1A). Additionally, in the presence of the regulatory subunits (2R2A, 2R2B, 2R2C) but not of the catalytic subunits (1CA, 2CA, 2CB), PAK6 appears phosphorylated (higher band* in the Phos-Tag gel, Figure 1, panel A). As active PAK6 is highly phosphorylated at S560 (autophosphorylation site) (Kaur et al., 2005) the presence of a higher, additional band may suggest that PP2A regulatory subunits stimulate PAK6 kinase activity. Next, using purified recombinant 3xFlag-PPP2R2C overexpressed with 2xMyc-PAK6 WT, PAK6-S531N or PAK6-K436M we performed a co-immunoprecipitation assay to verify that the kinase PAK6 binds PPP2R2C subunit. As reported in Figure 1B, we confirmed that PAK6 efficiently binds the PPP2R2C and that this binding is not affected by PAK6 kinase activity. To identify the exact site(s) at which PAK6 phosphorylates PPP2R2C, we then performed phospho-peptide enrichment followed by LC-MS/MS, as previously done for the identification of phospho-S59-14-3-3 as a PAK6 phosphorylation site (Civiero et al., 2017). These experiments identified S381 on PPP2R2C subunit as phosphorylated by PAK6 S531N but not by PAK6 K436M (Figure 1C). Of note, the amino acid sequence around S381 contains an Arginine (R) in position -2 which is typically present in bona fide type II PAKs substrates (Ha et al., 2015; Figure 1D). This phosphorylation site is positioned at the C-terminus of PPP2R2C (protein phosphatase 2, regulatory subunit B, gamma isoform a, NP_065149.2) and is conserved across vertebrates. It is worth noting that this site is also conserved in *Caenorhabditis elegans* (homologous gene F26E4.1), however, the overall low degree of evolutionary conservation in the adjacent aminoacids (Figure 1E), suggests that further research would be needed to verify a role for this residue in invertebrates. While a shift in the PhosTag gel is not evident for the other subunits, we identified additional putative PAK6 phosphosites in position Thr281 of PPP2CA and S120/Thr281 of PPP2CB catalytic subunits (Figure 1C). Together, these findings indicate that PAK6 phosphorylates in vitro the regulatory subunit PPP2R2C and, with lower stoichiometry, the catalytic subunits PPP2CA and PPP2CB.

### 3.2 PAK6-dependent phosphorylation of PPP2R2C does not affect PP2A holoenzyme formation

**FIGURE 2:**
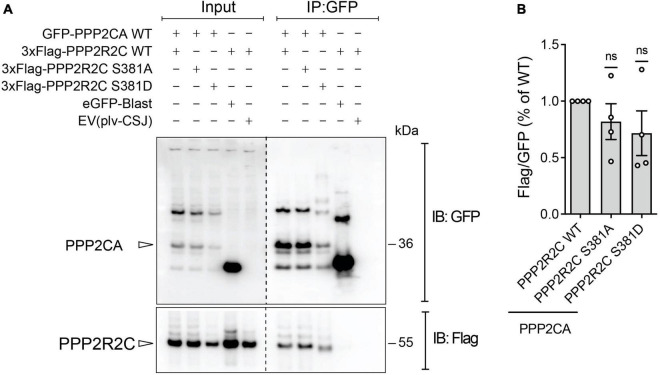
Assessment of PP2A holoenzyme formation with phosphomutant PPP2R2C at PAK6 dependent phosphosites. Co-immunoprecipitation between GFP-PPP2CA WT and 3xFlag-PPP2R2C WT or PAK6 phosphosite mutants (S381A and S381D). The immunoblot **(A)** and the relative quantification **(B)** show that mutation at PAK6 phosphosite does not impact on PP2A holoenzyme formation. (Data are expressed as mean ± SEM, *n* = 4 independent replicates, One sample *t*-test).

As the B regulatory subunit modulates substrate selectivity, subcellular localization and catalytic activity of PP2A (Mayer et al., 1991; Strack et al., 1998), we reasoned that phosphorylation of S381 by PAK6 may impact one or more of these properties. As the regulatory subunits regulates the enzyme through a direct binding with the catalytic core, we first assessed the effect of PAK6-mediated phosphorylation of S381 on PP2A holoenzyme formation. To this end, we performed a co-immunoprecipitation assay between GFP-PPP2CA wild-type and 3xFlag-PPP2R2C wild-type, S381A and S381D (phosphodeficient and phosphomimetic at PAK6 phosphosite, respectively). As shown in Figure 2, holoenzyme formation is not affected by the phosphorylation state of PPP2R2C-S381 (*n* = 4 independent replicates, *p* > 0.05, one sample *t*-test), suggesting that PAK6-mediated phosphorylation may affect other aspects of holoenzyme function. Importantly, as S381D single substitution results in a protein with decreased steady state levels, for the subsequent experiments we relied on the phosphodeficient mutant S381A.

### 3.3 The phosphorylation state of PPP2R2C at S381 regulates PPP2R2C subcellular localization

**FIGURE 3:**
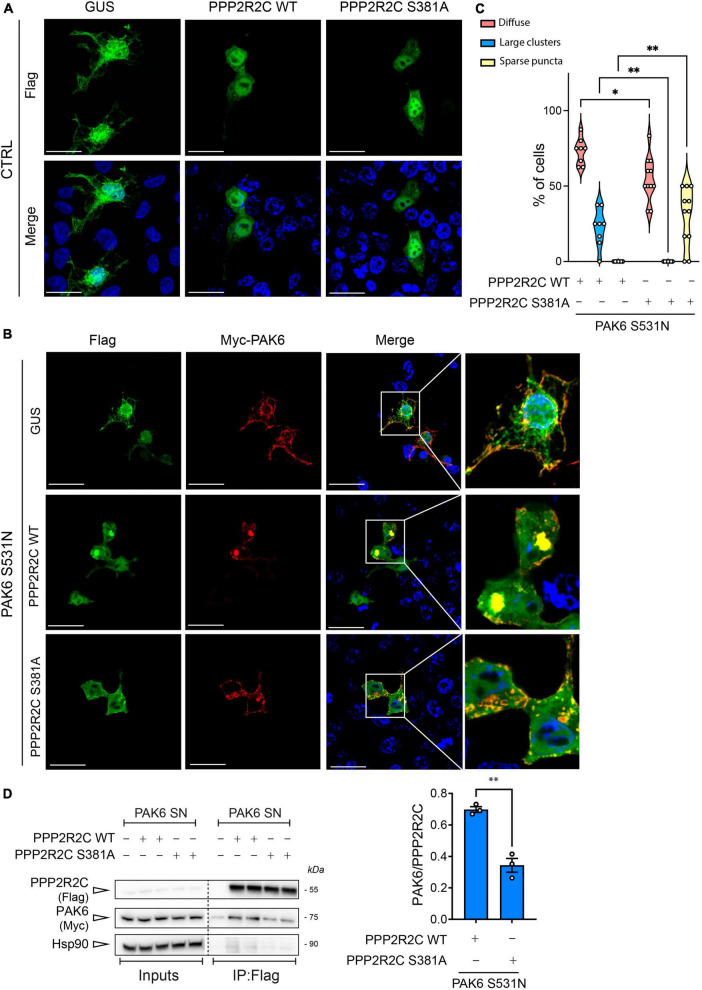
Evaluation of PAK6-mediated re-localization of PPP2R2C. **(A)** and **(B)** Representative images of transfected HEK293T cells showing re-localization of 3xFlag-GUS control or 3xFlag-PPP2R2C WT and SA in presence of PAK6 S531N (number of cells analyzed: PPP2R2C WT = 48, PPP2R2C WT + PAK6 S531N = 79, PPP2R2C S381A = 44, PPP2R2C S381A + PAK6 S531N = 67, *n* = 3 independent replicates, Two-way ANOVA with Tukey’s *post-hoc* test, *p = 0.001, **p < 0.005, Scale bar = 50 m). **(C)** Quantification of PPP2R2C re-localization in presence of PAK6 S531N. Western blot and relative quantification **(D)** of co-immunoprecipitation assay of 3xFlag-PPP2R2C WT or S381A and 2xMyc-PAK6 S531N. (*n* = 3 independent replicates, unpaired *t*-test, **p = 0.0018, data are expressed as mean ± SEM).

To investigate whether PPP2R2C-S381 phosphorylation impacts the subcellular localization of the enzyme, we next overexpressed 3xFlag-PPP2R2C WT and S381A in HEK293T cells and evaluated by immunocytochemistry PPP2R2C subcellular localization in the absence or presence of 2xMyc-PAK6 S531N, in order to maximize PAK6 phosphorylation of this site (Figure 1A). As shown in Figure 3, PPP2R2C is localized in the cytosol and nuclei and its localization is not affected by the aminoacid substitution S381A (Figure 3A, left panel). Instead, co-expression of PAK6 with PPP2R2C results in the re-localization of both proteins in clusters of variable size and number where the two enzymes co-localize (Figure 3B). In particular, PAK6 and PPP2R2C WT tend to form large perinuclear clusters (∼22% of co-transfected cells), whereas PAK6 and PPP2R2C S381A (phosphodeficient) mutant co-localize into smaller and sparse puncta (∼32% of co-transfected cells) (Figure 3C). These data indicate that PAK6 mediated phosphorylation of PPP2R2C at S381 influences the compartmentalization of the two proteins. To rule out that the effect of PAK6 S531N toward PPP2R2C is non-specific, we analyzed 3xFlag-GUS (a negative control used across our study) localization in the presence or absence of PAK6, confirming that PAK6 does induce 3xFlag-GUS re-localization into clusters (Figure 3B). Next, to evaluate whether a direct binding between PAK6 and PPP2R2C may be implicated in the regulation of their subcellular localization, we performed a co-immunoprecipitation of 3xFlag PPP2R2C WT or phosphodeficient (S381A) with 2xMyc-PAK6 S531N. As shown in Figure 3D, PAK6 interaction with PPP2R2C is negatively affected by the alanine substitution at S381, indicating that subcellular distribution of PAK6 PPP2R2C may also be affected by phosphorylation dependent binding of both proteins. Taken together, these data indicate a tight interplay between PAK6 and PPP2R2C affecting their interaction, phosphorylation, and subcellular localization.

### 3.4 PPP2R2C binding to LRRK2 is reduced when PPP2R2C is not phosphorylated by PAK6

**FIGURE 4:**
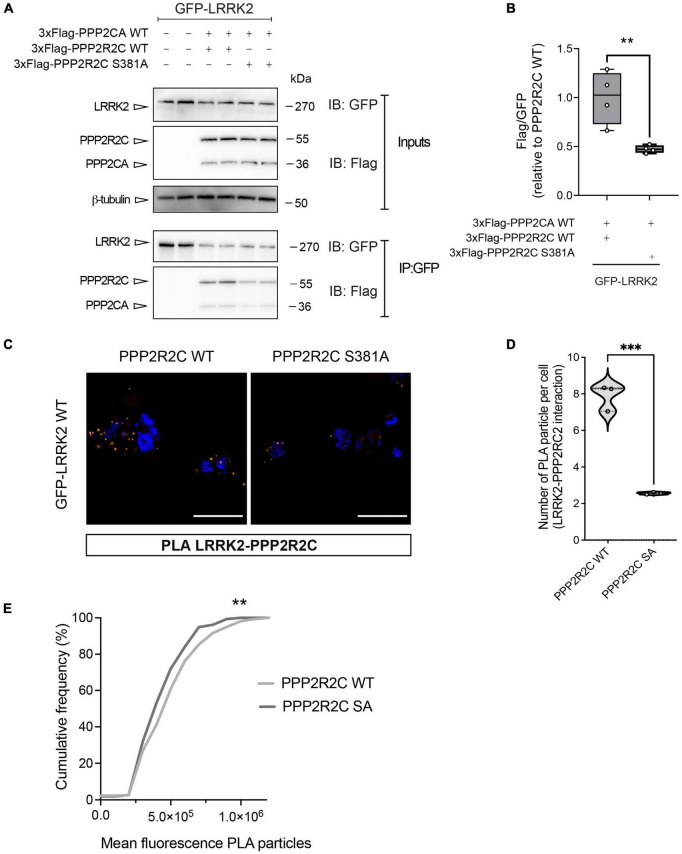
The non-phosphorylation of PPP2R2C at S381 negatively impacts its binding to LRRK2. Representative co-immunoprecipitation and subsequent western blot **(A)** and relative quantification **(B)** showing that the PPP2R2C phosphodead variant at its PAK6 site (S381A) is impaired in its binding with LRRK2 relative to PPP2R2C WT. (Data are expressed as mean ± SEM, Unpaired t test, **p = 0.0084, *n* = 4 independent replicates). **(C)** Representative confocal images of PLA between GFP-LRRK2 WT and Flag-PPP2R2C WT/S381A (scale bar = 25 m). **(D)** Number of PLA particles per cells (number of cells analyzed: LRRK2-PPP2R2C WT = 48, LRRK2-PPP2R2C S381A = 55, > 200 particles analyzed; *n* = 3 independent replicates; unpaired *t*-test; ***p < 0.0002). **(E)** Cumulative frequency distribution of particle fluorescence (Kolmogorov-Smirnov test, **p = 0.0017).

Since PAK6-dependent phosphorylation of PPP2R2C does not affect PP2A holoenzyme formation while influencing its subcellular localization and binding with PAK6, we next explored the possibility that PAK6 mediated-phosphorylation of PPP2R2C affects the interaction with its substrate LRRK2. To this end, we performed a co-immunoprecipitation assay between GFP-LRRK2 WT (IP) and 3xFlag PPP2R2C WT/S381A. As shown in Figures 4A, B, the binding between LRRK2 and PPP2R2C S381A is significantly reduced compared to PPP2R2C WT suggesting that PAK6-mediated phosphorylation at S381-PPP2R2C is important for the binding of the PP2A regulatory subunit with LRRK2. To confirm these findings with an independent approach, we performed Proximity Ligation Assay (PLA) in HEK293T cells co-transfected with GFP-LRRK2 WT and 3xFlag-PPP2R2C (WT or phosphodeficient S381A). A PLA signal is nicely detected when LRRK2 and PPP2R2C are co-transfected (Figure 4C). PLA signal is also present upon co-expression of GFP-LRRK2 WT and 3xFlag-PPP2CA although at a lower extent (data not shown). Instead, the number of PLA-positive puncta per cell and the mean fluorescence intensity of each puncta (panels E and F) are reduced in the presence of PPP2R2C S381A. Taken together, these results further support a mechanism whereby PAK6 phosphorylation of PPP2R2C at S381 modulates LRRK2:PPP2R2C interaction in cells (Figure 4).

### 3.5 Mutation of PPP2R2C at the PAK6 phosphosite affects LRRK2 phosphorylation levels at Ser935

**FIGURE 5:**
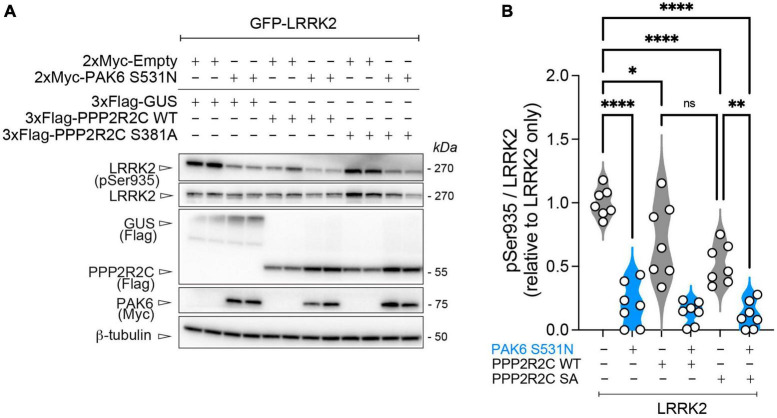
PAK6-mediated S381-PPP2R2C phosphorylation does not significantly impact PAK6 mediated dephosphorylation of LRRK2. **(A)** Representative western blot and **(B)** relative quantification of pS935-LRRK2/total LRRK2 in presence of PPP2R2C WT or S381A mutants (data are expressed as mean ± SEM, *n* = 7 independent replicates, One-way ANOVA followed by Tukey’s *post-hoc* test, *p = 0.0272, **p = 0.0013, ****p < 0.0001).

We recently reported that PP2A holoenzymes efficiently dephosphorylate LRRK2 at its phosphosites in the S935 cluster (Drouyer et al., 2021). Starting from these findings, we tested whether PAK6-dependent phosphorylation of PPP2R2C could affect LRRK2 phosphorylation levels when overexpressed. As shown in Figure 5, we observed that overexpression of PPP2R2C-S381A alone results in significantly reduced LRRK2 phosphorylation at S935 (*p < 0.05, *n* = 5 replicates, one-way ANOVA with Tukey’s *post-hoc* test). However, the effect of PPP2R2C S381A expression is modest compared to the effect of PAK6 S531N overexpression in inducing Ser935 dephosphorylation (Figure 5B). In addition, we tested whether these conditions affecting LRRK2 S935 phosphorylation could lead to changes in LRRK2 activity markers, including LRRK2 autophosphorylation at S1292 and phosphorylation of the LRRK2 substrate Rab10. The results shown in Supplementary Figure 3 indicate that LRRK2 activity markers are not significantly altered in the conditions tested.

## 4 Discussion

Mutations in the gene encoding for the multidomain protein LRRK2 can cause or predispose to PD (Rocha et al., 2022). Mutations in LRRK2 with confirmed pathogenicity can increase kinase activity (e.g., G2019S and I2020T) or result in a prolonged GTP-bound state (e.g., R1441C/G/H and Y1699C), with consequent increased access and phosphorylation of LRRK2 substrates (Greggio, 2012; Taymans, 2012; Iannotta and Greggio, 2021). LRRK2 is phosphorylated at multiple serine residues (including S910 and S935) between the ankyrin and the LRR domains, which play a central role in modulating LRRK2 activity and subcellular localization (Dzamko et al., 2010; Nichols et al., 2010; Marchand et al., 2020). Importantly, the majority of LRRK2 mutations result in reduced phosphorylation of this serine cluster (Nichols et al., 2010; Doggett et al., 2012; Marchand et al., 2020), underlying the importance of understanding the mechanisms that govern this post-translational modification. The phosphorylation cycle at this cluster of phosphosites is controlled by serine-threonine kinases (e.g., PKA, IKKs, and CK1) (Dzamko et al., 2012; Chia et al., 2014; Muda et al., 2014), but an important role is also played by phosphatases. We and other have previously reported that phosphatase subunits of the PP1 and PP2A phosphatase families dynamically bind and dephosphorylate LRRK2 (Lobbestael et al., 2013; Athanasopoulos et al., 2016; Drouyer et al., 2021). PP1 and PP2A are serine-threonine phosphatases organized as holoenzymes consisting of a catalytic subunit (PPP1CA/B/C and PPP2CA/B) and accessory subunits including a regulatory subunit, and in the case of PP2A also a scaffolding subunit (Cho and Xu, 2007). The presence of a large number of regulatory subunits (> 400 for PP1 and at least 24 for PP2A) (Braithwaite et al., 2012; Taymans and Baekelandt, 2014), reflects the need for providing high specificity of the phosphatase holoenzyme complex for the different substrates. While the search for PP1 regulatory subunits has remained elusive, we recently reported that PP2A holoenzymes with regulatory subunits PPP2R2A/B/C can effectively dephosphorylate LRRK2 (Drouyer et al., 2021). Interestingly, we have also previously shown that the LRRK2 interacting kinase PAK6 - by phosphorylating 14-3-3 – is responsible for LRRK2 dephosphorylation at the same phosphosite (Civiero et al., 2017). Whether PAK6 induces LRRK2 dephosphorylation also by affecting phosphatase activity is unknown.

To investigate this possibility, we performed *in vitro* phosphorylation using WT, constitutively active (S531N) and kinase dead (K436M) isoforms of PAK6 incubated with different subunits of PP1 and PP2A, reported to play a role in controlling LRRK2 phosphorylation state. Intriguingly, we found that PAK6 efficiently phosphorylates one of the PP2A regulatory subunits, PPP2R2C, which is exclusively expressed in the brain (Mayer et al., 1991; Strack et al., 1998; Janssens and Goris, 2001). The PAK6-mediated phosphorylation of PPP2R2C occurs at S381, a highly conserved residue among vertebrates (Figure 1, panel E). As the regulatory subunits are key modulators of PP2A holenzyme formation, substrate specificity, subcellular localization, and enzymatic activity (McCright et al., 1996; Leong et al., 2020), we decided to evaluate whether PPP2R2C phosphorylation at S381 could impact these PP2A features.

Holoenzyme assembly is tightly regulated in cells and occurs through the binding between a regulatory subunit B and the enzymatic core (AC) (Longin et al., 2004; Wepf et al., 2009; Brautigan and Shenolikar, 2018). As it has been reported that the enzyme assembly and the association with the regulatory subunits is regulated by phosphorylation and methyl-esterification of the catalytic subunit at specific sites (Tolstykh, 2000; Wei et al., 2001), it is reasonable to think that a phosphorylation of the regulatory subunits may also represent a mechanism to regulate the heterotrimeric enzyme association. However, we did not find evidence that the phosphorylation state of S381 plays a role in the formation of the active enzyme using a phosphomutant approach and co-immunoprecipitation (Figure 2). Therefore, as it has been reported that PPP2R2C localizes both in the nucleus and in the cytoplasm (McCright et al., 1996; Leong et al., 2020) we reasoned that PPP2R2C subcellular localization might be regulated by PAK6-mediated phosphorylation at S381. Intriguingly, the phosphorylation state of S381 does not impact PPP2R2C subcellular localization in basal conditions, as a phosphodead S381A variant of PPP2R2C showed unchanged subcellular localization compared to WT PPP2R2C (Figure 3). By contrast, in presence of active PAK6, PPP2R2C WT accumulates in unique large clusters while PPP2R2C S381A mutant preferentially accumulates in small clusters distributed in the cytoplasm (Figure 3). PPP2R2C has been reported to directly control multiple pathways in cells (reviewed in Amin et al., 2021), including regulating autophagy via dephosphorylation of both Ulk1 and Beclin1, respectively, promoting and downregulating the autophagic process (Wong et al., 2015; Fujiwara et al., 2016). Accordingly, in presence of PAK6, PPP2R2C accumulates in vesicular-like structures suggesting that PAK6 may regulate processes that require PPP2R2C translocation to vesicles. The different distribution pattern may also imply that PAK6 guides PPP2R2C to one or specific pools of substrates via S381 phosphorylation while it fails, at least partially, in re-localizing unphosphorylated PPP2R2C. The different localization pattern of PPP2R2C S381A in the presence or absence of PAK6 also suggests that PAK6 may control PPP2R2C localization with a kinase independent mechanism. Consistent with this is our finding that, PAK6 binds PPP2R2C and that this binding is significantly reduced in the presence of the S381A phosphodead mutant of PPP2R2C (Figure 3).

Previously, we demonstrated that the PPP2R2C homologs PPP2R2A and PPP2R2B bind LRRK2 and that this binding is enhanced when dephosphorylation of LRRK2 is induced by LRRK2 kinase inhibitors (Drouyer et al., 2021). Therefore, we also tested the involvement of PAK6-mediated phosphorylation of PPP2R2C in modulating the binding with LRRK2. This analysis revealed that the presence of the serine residue in the site 381 is important for the binding with LRRK2. Indeed, we observed an impairment of the binding of the PPP2R2C S381A phosphodead variant to LRRK2 compared to PPP2R2C WT (Figure 4). This observation may be explained by the PPP2R2C 3D structure visualized from AlphaFold (model reference AF-Q9Y2T4-F1, Figure 1, panel F) showing that S381 is located near the substrate binding region, represented by the central depression of the PPP2R2C toroid (Xu et al., 2008).

As we have previously shown that modulating expression of the PPP2R2C homolog PPP2R2A could lead to changes in LRRK2 S935 phosphorylation levels (Drouyer et al., 2021), we tested whether the S381A phosphodead PPP2R2C variant would also have an impaired ability to modulate LRRK2 phosphorylation. Surprisingly, we observed that overexpression of both the PPP2R2C WT the PPP2R2C-S381A led to a moderate but significant reduction in LRRK2 S935 phosphorylation rates, suggesting that non-phosphorylated PPP2R2C maintains the capacity to contribute to LRRK2 dephosphorylation despite being less readily recruited to the LRRK2 complex. Of note, expression of constitutively active PAK6 led to a more pronounced dephosphorylation of LRRK2 at S935 than PPP2R2C WT/SA expression alone and the PAK6 mediated dephosphorylation of LRRK2 was not significantly affected by the presence of PPP2R2C WT or S381A variants (Figure 5). Accessorily, in these conditions leading to reduced LRRK2 phosphorylation were tested for their impact on LRRK2 activity using LRRK2 autophosphorylation and endogenous phosphorylation of the LRRK2 substrate Rab10 as readouts. The results (Supplementary Figure 3) do not show significant changes in LRRK2 activity in these conditions, in line with our recent observations that LRRK2 phospho-dead variants do not alter LRRK2 kinase activity under basal conditions. Further work will be required to address this question, in particular testing the LRRK2-PAK6-PPP2R2C complex in activation conditions and in the presence of direct modulators of the complex.

**FIGURE 6:**
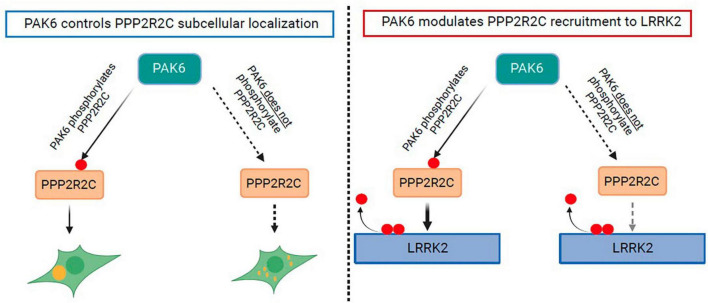
Schematic of effects of PAK6 mediated phosphorylation of PPP2R2C. Effects are depicted of PAK6 mediated phosphorylation of PPP2R2C on its subcellular localization (left panel) and on its recruitment to the LRRK2 complex (right panel).

The observations on LRRK2 dephosphorylation in the presence of both PAK6 and PPP2R2C allow us to refine a proposed mechanism for PAK6 mediated dephosphorylation of LRRK2 at the S935 cluster (visually summarized in Figure 6). At least some of the phosphosites at this cluster, including S910 and S935, are known to bind 14-3-3 dimers that may act as a protective cap preventing dephosphorylation by phosphatases (Nichols et al., 2010; Stevers et al., 2017; Manschwetus et al., 2020). Previously, we found that PAK6 can phosphorylate 14-3-3γ, thereby disrupting the 14-3-3 dimer that is the released from the LRRK2 phosphosites, exposing them to dephosphorylation by phosphatases (Civiero et al., 2017). In the present paper, we have found that PAK6 in addition phosphorylates PPP2R2C and that this modulates PPP2R2C recruitment to the LRRK2 complex without affecting PP2A holoenzyme formation. Together with the modest effect of PPP2R2C overexpression on LRRK2 dephosphorylation, this suggests that the crucial step in the PAK6 mechanism of LRRK2 dephosphorylation remains the uncapping of the LRRK2 phosphosites through PAK6 mediated phosphorylation of 14-3-3γ. Our results indicate that there is an additional layer of fine tuning of the LRRK2 dephosphorylation mechanism via regulation of PPP2R2C recruitment to LRRK2 by a PAK6 dependent phosphorylation. Given the hypothesis that LRRK2 dephosphorylation is associated to disease states and that PPP2R2C is a brain-enriched protein, further work is warranted to explore the PAK6:PPP2R2C:14-3-3 mechanisms of LRRK2 dephosphorylation in brain and disease models.

## Data availability statement

The data presented in the study are deposited in the ProteomeXchange repository, accession number PXD044933.

## Ethics statement

Ethical approval was not required for the studies on humans in accordance with the local legislation and institutional requirements because only commercially available established cell lines were used.

## Author contributions

LI: Data curation, Formal analysis, Writing−original draft, Writing−review and editing. ME: Data curation, Formal analysis, Writing−review and editing. GT: Data curation, Writing−review and editing. LV: Data curation, Formal analysis, Writing−review and editing. ALO: Writing−review and editing. J-MS: Data curation, Writing−review and editing. MD: Writing−review and editing. WS: Data curation, Writing−review and editing. LC: Writing−review and editing. RJN: Writing−review and editing. PA: Writing−review and editing. AK: Writing−review and editing. M-CC-H: Writing−review and editing. EG: Conceptualization, Funding acquisition, Project administration, Resources, Supervision, Writing−original draft, Writing−review and editing. J-MT: Conceptualization, Data curation, Funding acquisition, Resources, Writing−original draft, Writing−review and editing. GF: Data curation, Writing−review and editing.
