# Supplementary material for: PAK6-mediated phosphorylation of PPP2R2C regulates LRRK2-PP2A complex formation
Source: Front Mol Neurosci. 2023 Dec 18;16:1269387. doi: 10.3389/fnmol.2023.1269387 (PMC10759229; doi:10.3389/fnmol.2023.1269387)
Supplement: Supplementary file 1 [file Data_Sheet_1.docx]

Supplementary Material

PAK6-mediated phosphorylation of PPP2R2C regulates LRRK2-PP2A complex formation without affecting PP2A activity

Lucia Iannotta^1,2^, Marco Emanuele^3^, Giulia Favetta^1^, Giulia Tombesi^1,4^, Laurine Vandewynckel^3^, Antonio Jesús Lara-Ordóñez^3^, Jean-Michel Saliou^5^, Matthieu Drouyer^3^, William Sibran^3^, Laura Civiero^1,6,7^, Jeremy Nichols^8^, Panagiotis Athanasopoulos^9^, Arjan Kortholt^9^, Marie-Christine Chartier-Harlin^3^, Elisa Greggio^1,7, †,*^ and Jean-Marc Taymans^3, †,*^

^1^ University of Padova, Department of Biology, Padova, Italy; [lucia.iannotta@unipd.it](mailto:lucia.iannotta@unipd.it);

^2^ Institute of Neuroscience, National Research Council, c/o Humanitas Research Hospital, 20089 Rozzano, Milan, Italy; [lucia.iannotta@humanitasresearch.it](mailto:lucia.iannotta@humanitasresearch.it);

^3^ Univ. Lille, Inserm, CHU Lille, UMR-S 1172 - LilNCog - Lille Neuroscience & Cognition, F-59000 Lille, France;

^4^ Department of Pharmacology, Feinberg School of Medicine, Northwestern University, Chicago, IL, USA;

^5^ Univ. Lille, CNRS, Inserm, CHU Lille, Institut Pasteur de Lille, US 41 - UAR 2014 - PLBS, F-59000 Lille, France;

^6^ IRCSS, San Camillo Hospital, Venice, Italy;

^7^ Centro Studi per la Neurodegenerazione (CESNE), University of Padova, Italy.

^8^ Department of Pathology, Stanford University, 300 Pasteur Drive, Stanford, CA 94305, USA.

^9^ Department of Cell Biochemistry, University of Groningen, Groningen, Netherlands

^†^ These authors contributed equally to this work and share last authorship

*** Correspondence:**Elisa Greggio [elisa.greggio@unipd.it](mailto:elisa.greggio@unipd.it) and Jean-Marc Taymans [jean-marc.taymans@univ-lille.fr](mailto:jean-marc.taymans@univ-lille.fr)

# Supplementary Figures


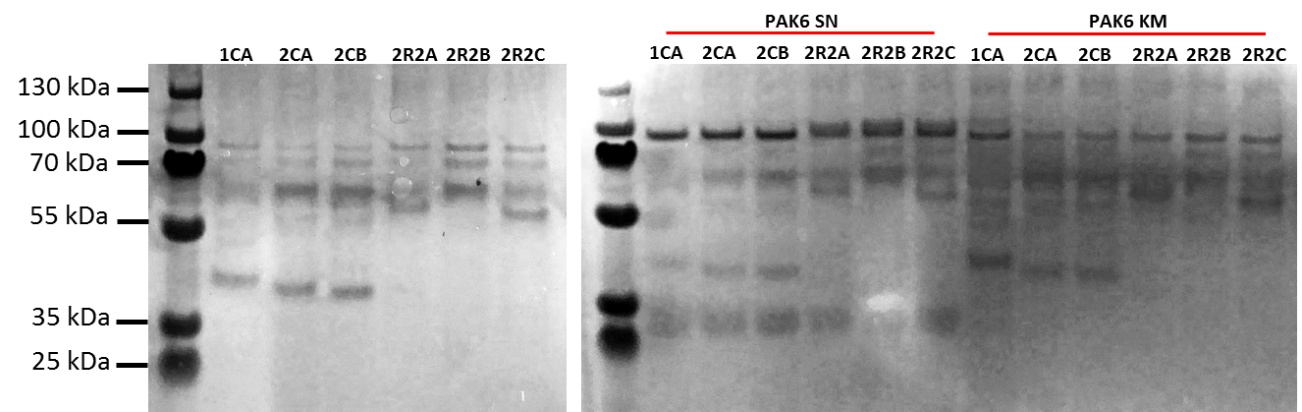


**Supplementary Figure 1.** Coomassie brilliant blue staining of SDS-PAGE gels of purified phosphatases (left panel) and the same phosphatases together with hyperactive (SN) and kinase dead (KM) PAK6 (right panel). PAK6 band is found at approximately 75 kDa, while sizes of phosphatases are 38 kDa for PPP1CA, 36 kDa for PPP2CA and PPP2CB, 55 kDa for PPP2R2A and PPP2R2C, 60 kDa for PPP2R2B.


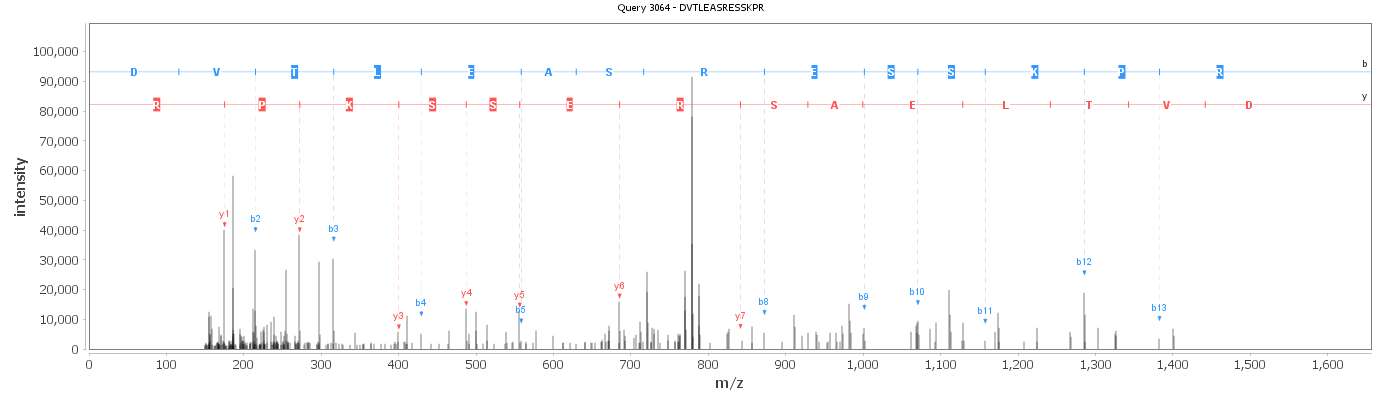


**Supplementary Figure 2.** MS/MS analysis of the PPP2R2C protein that was submitted to PAK6 phosphorylation (see Figure 1). Shown is the MS/MS spectrum of PPP2R2C peptide 372-385 that displays phosphorylation at Ser381 of PPP2R2C (m:z=827,9864, ion score 52.4).


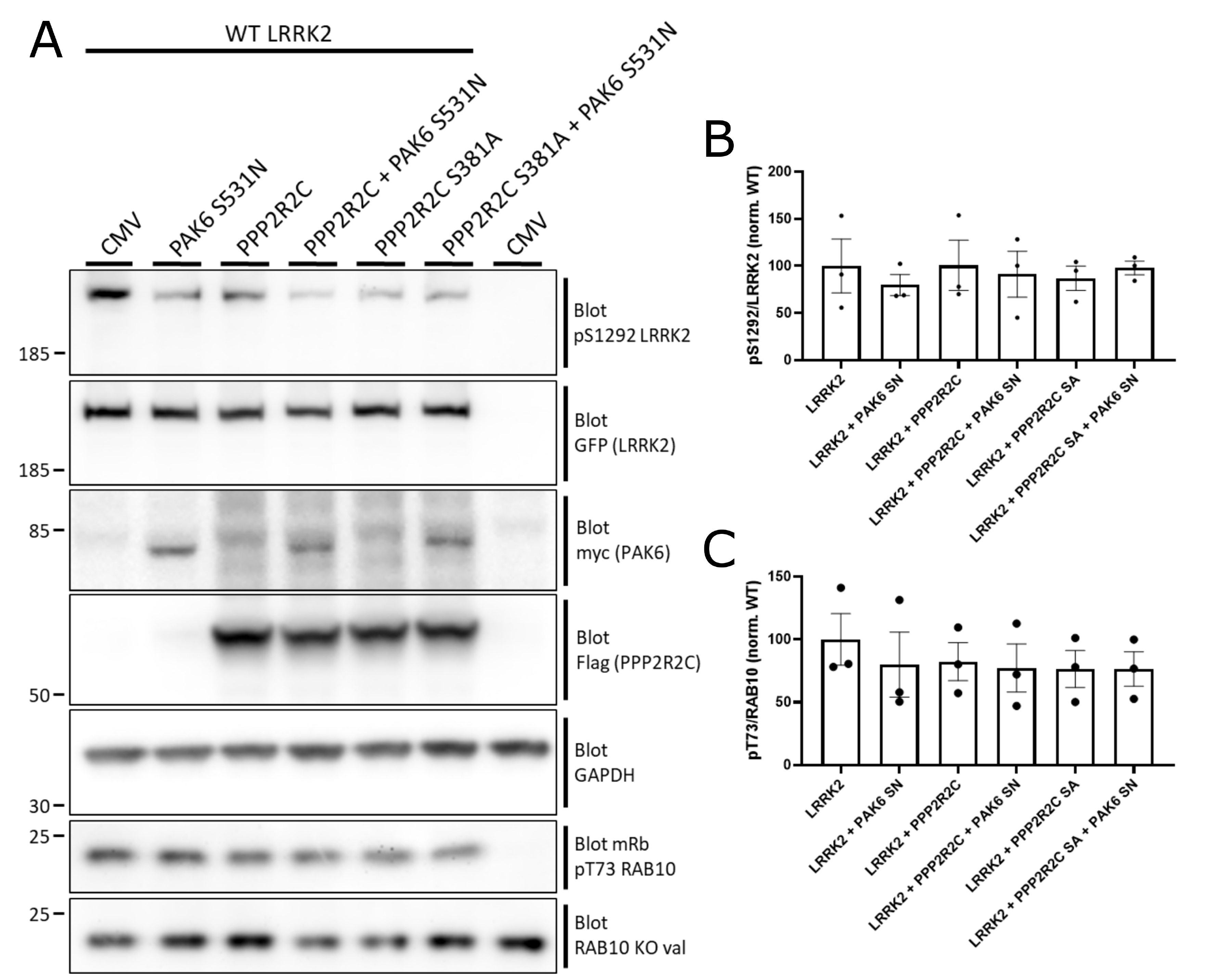


**Supplementary Figure 3.** Assessment of LRRK2 activity markers upon expression of PPP2R2C and PAK6. Experimental conditions that lead to alterations in LRRK2 S935 phosphorylation, equivalent to those presented in Figure 5, were tested via western blot for their effects on LRRK2 activity markers including LRRK2 autophosphorylation at S1292 and phosphorylation of the LRRK2 substrate Rab10. Conditions include overexpression of the constitutively active PAK6 S531N variant as well as PPP2R2C WT or the non-phosphorylatable S381A variant and combinations thereof, using CMV empty vector as controls. Representative blot images are given in (A), quantification of the blots are given in (B) for pS1292-LRRK2 and (C) for pT73-Rab10. Experiments were performed in triplicate and measured in 2 technical replicates. Quantifications and statistical analysis (ANOVA with post-hoc Dunnet’s test using LRRK2 WT as control condition) yielded no significant differences between the different experimental groups in the conditions tested.
